# Supplementary material for: Association of albumin, fibrinogen, and modified proteins with acute coronary syndrome
Source: PLoS One. 2022 Jul 26;17(7):e0271882. doi: 10.1371/journal.pone.0271882 (PMC9321412; doi:10.1371/journal.pone.0271882)
Supplement: Attachment — Submitted filename: Response to Reviewers.docx [file pone.0271882.s001.docx]

Journal Requirements:

When submitting your revision, we need you to address these additional requirements.

1. Please ensure that your manuscript meets PLOS ONE's style requirements, including those for file naming. The PLOS ONE style templates can be found at

<https://journals.plos.org/plosone/s/file?id=wjVg/PLOSOne_formatting_sample_main_body.pdf> and

<https://journals.plos.org/plosone/s/file?id=ba62/PLOSOne_formatting_sample_title_authors_affiliations.pdf>

Response: We ensure that our manuscript meets PLOS ONE’s style requirements, including those for file naming.

2. Thank you for stating the following financial disclosure:

“This study was funded by a research grant awarded to Dr. LNI by the Ministry of Science and Technology, Government of the People’s Republic of Bangladesh.”

Please state what role the funders took in the study.  If the funders had no role, please state: "The funders had no role in study design, data collection and analysis, decision to publish, or preparation of the manuscript."

If this statement is not correct you must amend it as needed.

Please include this amended Role of Funder statement in your cover letter; we will change the online submission form on your behalf.

Response: In the cover letter, the following statement has been included "The funders had no role in study design, data collection and analysis, decision to publish, or preparation of the manuscript."

Additional Editor Comments:

Please edit the manuscript carefully as suggested by the reviewers.

Check reference style, word count by consulting the instructions for authors.

[Note: HTML markup is below. Please do not edit.]

Reviewers' comments:

Reviewer's Responses to Questions

Comments to the Author

1. Is the manuscript technically sound, and do the data support the conclusions?

The manuscript must describe a technically sound piece of scientific research with data that supports the conclusions. Experiments must have been conducted rigorously, with appropriate controls, replication, and sample sizes. The conclusions must be drawn appropriately based on the data presented.

Reviewer #1: Yes

Reviewer #2: Partly

Reviewer #3: Partly

2. Has the statistical analysis been performed appropriately and rigorously?

Reviewer #1: Yes

Reviewer #2: Yes

Reviewer #3: Yes

3. Have the authors made all data underlying the findings in their manuscript fully available?

The PLOS Data policy requires authors to make all data underlying the findings described in their manuscript fully available without restriction, with rare exception (please refer to the Data Availability Statement in the manuscript PDF file). The data should be provided as part of the manuscript or its supporting information, or deposited to a public repository. For example, in addition to summary statistics, the data points behind means, medians and variance measures should be available. If there are restrictions on publicly sharing data—e.g. participant privacy or use of data from a third party—those must be specified.

Reviewer #1: Yes

Reviewer #2: Yes

Reviewer #3: Yes

4. Is the manuscript presented in an intelligible fashion and written in standard English?

PLOS ONE does not copyedit accepted manuscripts, so the language in submitted articles must be clear, correct, and unambiguous. Any typographical or grammatical errors should be corrected at revision, so please note any specific errors here.

Reviewer #1: Yes

Reviewer #2: No

Reviewer #3: Yes

5. Review Comments to the Author

Please use the space provided to explain your answers to the questions above. You may also include additional comments for the author, including concerns about dual publication, research ethics, or publication ethics. (Please upload your review as an attachment if it exceeds 20,000 characters)

Reviewer #1: The study is interesting and quite well written. The conclusions, if confirmed by larger studies, could have clinical relevance. The main limitation, as pointed out by the authors, is the low sample size.

This reviewer raises some issues that need to be addressed by the authors.

1- The authors included diabetes among the exclusion criteria. However, during ACS, hyperglycemia is also frequently found in non-diabetic individuals. In this setting, tight glycemic control favorably influences the CV outcomes of these patients (Journal of Clinical Endocrinology and Metabolism Volume 97, Issue 3, March 2012, 933-942. doi: 10.1210/jc.2011-2037 - Journal of Diabetes Research, 2018, art. no. 3106056. doi: 10.1155/2018/3106056). The text and tables are missing glucose values during hospitalization for ACS, which should have been included in the multivariate analysis. This issue, which is a limitation of the study, and the above references should be discussed in the manuscript.

Reply to the reviewer: As mentioned by the learned reviewer, the stated references observed that tight glycemic control may increase regenerative potential of the ischemic myocardium (J Clin Endocrinol Metab. 2012; 97:933-942, doi: 10.1210/jc.2011-2037), and hyperglycemia is common during ACS which is a significant and independent mortality predictor among diabetic patients with recent ACS (J Diabetes Res. 2018, art. no. 3106056.  doi: 10.1155/2018/3106056). Considering these, we excluded patients and controls with hyperglycemic random blood sugar levels from our study, so that the association of albumin, fibrinogen and modified proteins with ACS could be assessed independently from hyperglycemia. It should be mentioned here that all patients enrolled in the study had blood glucose levels below 6.5 mmol/L on admission.

2- In multivariate logistic regression analyzes, age and hypertension are not independently correlated with ACS. How do the authors justify these unexpected results?

Reply to the reviewer: To determine the potential of albumin, fibrinogen and other baseline characteristics in predicting risk of ACS, both univariate (not shown in the manuscript) and multivariate logistic regression analyses were conducted. In univariate logistic regression, age, hypertension, family history of CVD, albumin and fibrinogen all were independent risk factors for ACS. Upon multivariate logistic regression analysis, age and hypertension were found to be not significant with respect to all other parameters.

Reviewer #2: The authors conducted “Assessing Circulatory Proteins in Patients with ACS”. While the idea is interesting, I have several concerns.

• The major weakness of this article is that extensive editing of the English language and style is required. Indeed, some parts are absolutely not comprehensible.

Reply to the reviewer: We have edited the Introduction, Materials and Methods, and Discussion sections to make them more comprehensible. We believe, the learned reviewer would find an overall improvement of language in the Revised Manuscript.

• The article’s main title is ambiguous and should be rephrased to be consistent with the precise goal.

Reply to the reviewer: The title of the article has been rephrased to be consistent with the precise goal of the study:

Association of albumin, fibrinogen, and modified proteins with acute coronary syndrome

 • Line 55-58: Please indicate the source of this text.

Reply to the reviewer: A reference has been added in the manuscript to indicate the source of the text.

• Line 59-61: This text does not appear to be in keeping with the preceding text's context, please find sources that narrate the dangerous events of this disease

Reply to the reviewer: This has been addressed in the manuscript.

• Please pay attention to writing the main and sub-headings of the article, and follow the basic approaches to writing scientific research

Reply to the reviewer: The main and sub-headings of the article have been changed and improved.

• Line 100: Please change the title “Subjects and methods” to “Materials and Methods”

Reply to the reviewer: The title has been changed in the manuscript.

• How was the sample size calculated, and is the number of specific subjects sufficient for this study, please explain it in detail.

Reply to the reviewer: Acute coronary syndrome (ACS, formerly called ischemic heart disease) is caused by a sudden onset of cardiac tissue ischemia secondary to impaired blood flow. Bangladeshi people have high susceptibility to ischemic heart disease (IHD) although no population-based data is available. One study found the prevalence of IHD in Bangladeshi men to be 4.6% (Zaman et al, 2007; PMID: 19124932). It may be mentioned here that all our study participants were men, which had been inadvertently omitted from the manuscript. It has now been mentioned under the heading “Subjects” in the manuscript. Therefore considering the available prevalence rate, and owing to the difficulties in collecting blood samples from patients admitted to the CCU by following the stringent inclusion criteria of this study, we had to consider a single parameter (namely, human serum albumin) to calculate the sample size. The formula used is:

n = Z^2^p(1-p)/d^2^, where n = sample size; Z = confidence level at 95%; p = expected prevalence (here, 4.6%); and d = precision (±5%).

By substituting all values in the formula, we get:

n = 1.96^2^×0.046(1-0.046)/0.05^2^ = 67.4

Thus, we collected 70 samples from ACS patients.

Please note that this number is not sufficient as there are other parameters in this study. We have mentioned this in the limitations of our study.

• Why did you specify the ages of the participants from 30 to 70 years? If you mean that the participants were within the ages you specified, then you must transfer this information to the results section and include the acceptable ages in the study.

Reply to the reviewer: In a recent study carried out in Bangladesh on 800 hospitalized patients with ACS, it was found that approximately 5% of patients were less than 30 years of age while about 10% were aged 70 years or more (Ahmed et al., 2018; doi.org/:[10.3329/bhj.v33i1.37018](http://dx.doi.org/10.3329/bhj.v33i1.37018)). We found a similar scenario in our preliminary study; hence we selected this age range to enroll study participants.

• The inclusion and exclusion criteria are confusing and unclear.

Reply to the reviewer: We have revised the relevant section and clarified the inclusion and exclusion criteria of the study subjects in the manuscript.

• Please add a paragraph entitled Study Procedures, and explain all the details of the study to be clear to the reader

Reply to the reviewer: We have added a paragraph entitled Study Procedures, and explained all the details of the study to be clear to the reader.

• Please delete the "Study period and blood sample collection" paragraph and move the information about it to the study procedures paragraph

Reply to the reviewer: We have provided the information on study period and blood sample collection to the study procedures paragraph.

• Please specify the end points of the study in a separate paragraph

Reply to the reviewer: Since our study was a case-control study, we did not specify any end points. In future similar observational cohort studies or clinical trials, the study end points could include all cause mortality, recurrent MI, cardiovascular deaths, stroke, etc.

• Put all the basic tests under the main title "Measurements"

Reply to the reviewer: All the basic tests have been put under the heading “Study procedures”, as suggested by Reviewer #3 (point no. 2, below).

• In table 1: change “ACS Cases” to “ACS group”, “Controls” to “Control group”, and “Statistics” to “p-value. Also, please find the P-values for BMI, SBP, and DBP and add them to the table.

Reply to the reviewer: All suggestions of the learned reviewer have been incorporated in Table 1 of the revised manuscript.

• Line 167: Cheng the title “Level of serum albumin in the study subjects” to “level of human serum albumin”, and please illustrate the results of this test with a graph.

Reply to the reviewer: The change has been made in the manuscript. The results of human serum albumin in the ACS and Control groups have been illustrated with a figure (Fig 1).

• finally, this study has several limitations that may affect the results of the study.

Reply to the reviewer: The limitations of this study have been discussed in the manuscript. However, the conclusions, if confirmed by larger studies, could have clinical relevance.

Reviewer #3: Nabila Nawar Binti, et al. demonstrate that Albumin, ischemia modified albumin (IMA) and protein carbonyls were found to have high diagnostic sensitivity and specificity for ACS. Of interest, these circulatory and modified proteins in ACS patients, particularly lower HSA, AGR, and higher IMA and protein carbonyls showed the potentials to be used for risk assessment of ACS.

The study is of interest nevertheless, I have several concerns that need to be addressed before the study could be re-submitted.

1) Introduction: please, shorten the introduction focusing on the biomarkers.

Reply to the reviewer: The introduction has been shortened and focused on the biomarkers.

2) Methods: Please merge the paragraphs from 2.4 to 2.10 in one single session about the Labo test of the biomarkers/circulating proteins.

Reply to the reviewer: The paragraphs 2.4 to 2.10 have been merged in one single session under the heading “Study procedures”.

3) Methods: please describe how family history of CVD was assessed considering that is one of the independent factors of the multivariate logistic regression.

Reply to the reviewer: Details have been added in the manuscript under the heading “Study Procedures”. Briefly - in a carefully pre-designed questionnaire, all the general information for each study subject was recorded which included their age, height, weight, blood pressure, any family history of CVD, and hypertension.

4) Results: “The duration of chest pain, from onset to hospitalization of the patients varied 158 from 0.5 to 120 hours, with a median of 6.0 hours”. Please, split the symptoms to balloon time for STEMI and NSTEMI/UA.

Reply to the reviewer: Unfortunately, this information was not available from the hospital.

5) Results: dyslipidemia, previous history of CAD and admission medical therapy should be added to table 1 in order to better describe the 2 study populations.

Reply to the reviewer: The lipid profile of the study participants was measured in the study but not included in the manuscript. There were no significant differences in serum levels of cholesterol, triglycerides, and LDL cholesterol among the two study populations (it should be mentioned here that the patients were already hospitalized and under cholesterol-lowering drugs). However, the HDL cholesterol was found significantly diminished in the patient group in contrast to that of the control group. The previous history of the patients showed 9 had MI, 5 had angina, and 5 suffered cardiac arrest (this information has been added in the manuscript under the heading “Baseline features of the study participants”). The admission medical therapy data of the patients was unavailable.

6) Figure Legends: I am not sure that the figure legend should be in the text. Please, verify.

Reply to the reviewer: The PLOS ONE style template has been checked regarding the placement of the figure legend.

7) Results: Please merge the paragraphs 3.4 - 3.5 and 3.6 – 3.7 in two different paragraphs.

Reply to the reviewer: This has been addressed in the manuscript.

8) Results: Please add some information regarding the angiographic data (vessels affected by the lesions, PCI performed, numbers of stent).

Reply to the reviewer: Unfortunately, the suggested information was not available from the hospital.

9) Results: were there any patients without significant coronary artery disease (MINOCA)? Please clarify this information.

Reply to the reviewer: Clinicians did not report any such observation.

10) Results: Please, can the authors provide some data about the standard inflammatory agents such as WBC (white blood cells), neutrophils and lymphocytes counts and CRP.

Reply to the reviewer: We have done the blood platelet count (data not shown) and measured fibrinogen levels in plasma of the study participants and found both the inflammatory parameters to be significantly higher in the ACS group. However, in a recently published paper from our lab, the WBC count of ACS patients was found to be 11.76 ± 2.49 × million cells/mL compared to 7.37 ± 1.77 × million cells/mL in the control subjects (p<0.001); the neutrophil count was significantly higher and lymphocyte count was significantly lower in patients (Afr.J.Bio.Sc. 4(1) (2022) 37-47). In the present study, some of the patients had CRP level between 6.0 and 20.0 mg/L.

11) Results: Please, can the authors provide some data about the admission blood glucose level and possible correlations with these circulating inflammatory proteins (albumin, ischemia modified albumin (IMA) and protein carbonyls)?

Reply to the reviewer: All patients enrolled in the study had blood glucose levels below 6.5 mmol/L on admission. Individual values were not recorded for further analysis.

12) Discussion: Please shorten the discussion, focusing on the main findings.

Reply to the reviewer: We have shortened the discussion, focusing on the main findings.

13) Discussion: Please integrate the discussion with the following ref PMID: 33530978 regarding the inflammatory burden in patients with acute myocardial infarction.

Reply to the reviewer: Unfortunately, we couldn’t integrate the discussion with the suggested reference (PMID 33530978) since the study focused on the interplay between hyperglycemia, inflammation and infarct size in a cohort of patients admitted with acute myocardial infarction, including cases of MINOCA, which did not exactly relate to our study.

6. PLOS authors have the option to publish the peer review history of their article (what does this mean?). If published, this will include your full peer review and any attached files.

If you choose “no”, your identity will remain anonymous but your review may still be made public.

Do you want your identity to be public for this peer review? For information about this choice, including consent withdrawal, please see our Privacy Policy.

Reviewer #1: No

Reviewer #2: No

Reviewer #3: No

[NOTE: If reviewer comments were submitted as an attachment file, they will be attached to this email and accessible via the submission site. Please log into your account, locate the manuscript record, and check for the action link "View Attachments". If this link does not appear, there are no attachment files.]

While revising your submission, please upload your figure files to the Preflight Analysis and Conversion Engine (PACE) digital diagnostic tool, https://pacev2.apexcovantage.com/. PACE helps ensure that figures meet PLOS requirements. To use PACE, you must first register as a user. Registration is free. Then, login and navigate to the UPLOAD tab, where you will find detailed instructions on how to use the tool. If you encounter any issues or have any questions when using PACE, please email PLOS at figures@plos.org. Please note that Supporting Information files do not need this step.

Response: We uploaded our figure files (a total of 5 figures) to the PACE digital diagnostic tool to check whether the figures met PLOS requirements; all figures were converted to the accepted formats of PLOS ONE.
