# Supplementary material for: Association of albumin, fibrinogen, and modified proteins with acute coronary syndrome
Source: PLoS One. 2022 Jul 26;17(7):e0271882. doi: 10.1371/journal.pone.0271882 (PMC9321412; doi:10.1371/journal.pone.0271882)
Supplement: Attachment — Submitted filename: Response to Reviewers.docx [file pone.0271882.s002.docx]

PONE-D-21-36646R1

Association of albumin, fibrinogen, and modified proteins with acute coronary syndrome

Journal Requirements:

Please review your reference list to ensure that it is complete and correct. If you have cited papers that have been retracted, please include the rationale for doing so in the manuscript text, or remove these references and replace them with relevant current references. Any changes to the reference list should be mentioned in the rebuttal letter that accompanies your revised manuscript. If you need to cite a retracted article, indicate the article’s retracted status in the References list and also include a citation and full reference for the retraction notice.

Response: We have reviewed our reference list to ensure that it is complete and correct.

Reference numbers 17 and 25 have been newly inserted at line # 111 and line # 246, respectively. Please note that these references have been included as per suggestions of the learned Reviewer #1.

The following references have been added to the reference list in the manuscript:

17.	Sasso FC, Rinaldi L, Lascar N, Marrone A, Pafundi PC, Adinolfi LE, Marfella R (2018) Role of tight glycemic control during acute coronary syndrome on CV outcome in type 2 diabetes. J Diabetes Res 3106056.

25.	Marfella R, Sasso FC, Cacciapuoti F, Portoghese M, Rizzo MR, Siniscalchi M et al. (2012) Tight glycemic control may increase regenerative potential of myocardium during acute infarction. J Clin Endocrinol Metab 97(3):933-42.

Additional Editor Comments (if provided):

You are invited to consider the reviewers' comments, reported at the end of this letter, and to revise your manuscript accordingly. In the letter accompanying your resubmission, please explain your response to each of the comments. Please observe the word count and citation style. For further details, please consult the Instructions for Authors on the website

Response: During resubmission of the previous version of our manuscript [PONE-D-21-36646], we have answered to the first question made by Reviewer #1 in our ‘Response to Reviewers’, which may not have been adequate, and the suggested references have not been included by mistake. We apologize for the inadvertent omissions. In the accompanying revised version of our manuscript [PONE-D-21-36646R1], we have tried to address the issue in detail with the inclusion of the references. Regarding the second question, we are apologetic for failing to clarify the second issue both in our manuscript and ‘Response to Reviewers’. We have included an extra column in Table 2 in the accompanying revised version of our manuscript [PONE-D-21-36646R1], to present the statistical findings of the univariate logistic regression analyses along with those of the multivariate regression data. We found age and hypertension both to be significant risk factor for ACS when each was considered independently; however, the multivariate logistic regression analyses found none of these to be significant risk factors after adjusting for more important biochemical parameters such as albumin and fibrinogen. We hope our point would be clear this time.

[Note: HTML markup is below. Please do not edit.]

Reviewers' comments:

Reviewer's Responses to Questions

Comments to the Author

1. If the authors have adequately addressed your comments raised in a previous round of review and you feel that this manuscript is now acceptable for publication, you may indicate that here to bypass the “Comments to the Author” section, enter your conflict of interest statement in the “Confidential to Editor” section, and submit your "Accept" recommendation.

Reviewer #1: (No Response)

Reviewer #2: All comments have been addressed

Reviewer #3: All comments have been addressed

2. Is the manuscript technically sound, and do the data support the conclusions?

The manuscript must describe a technically sound piece of scientific research with data that supports the conclusions. Experiments must have been conducted rigorously, with appropriate controls, replication, and sample sizes. The conclusions must be drawn appropriately based on the data presented.

Reviewer #1: Yes

Reviewer #2: Yes

Reviewer #3: Partly

3. Has the statistical analysis been performed appropriately and rigorously?

Reviewer #1: Yes

Reviewer #2: Yes

Reviewer #3: Yes

4. Have the authors made all data underlying the findings in their manuscript fully available?

The PLOS Data policy requires authors to make all data underlying the findings described in their manuscript fully available without restriction, with rare exception (please refer to the Data Availability Statement in the manuscript PDF file). The data should be provided as part of the manuscript or its supporting information, or deposited to a public repository. For example, in addition to summary statistics, the data points behind means, medians and variance measures should be available. If there are restrictions on publicly sharing data—e.g. participant privacy or use of data from a third party—those must be specified.

Reviewer #1: Yes

Reviewer #2: Yes

Reviewer #3: Yes

5. Is the manuscript presented in an intelligible fashion and written in standard English?

PLOS ONE does not copyedit accepted manuscripts, so the language in submitted articles must be clear, correct, and unambiguous. Any typographical or grammatical errors should be corrected at revision, so please note any specific errors here.

Reviewer #1: Yes

Reviewer #2: Yes

,

Reviewer #3: Yes

6. Review Comments to the Author

Please use the space provided to explain your answers to the questions above. You may also include additional comments for the author, including concerns about dual publication, research ethics, or publication ethics. (Please upload your review as an attachment if it exceeds 20,000 characters)

Reviewer #1: The authors did not address the two issues I raised.

Specifically, regarding the first question, they made a comment in their answer but did not include that comment or the references suggested in the manuscript.

As for the second question, they did not address it either in the answer or in the manuscript.

Therefore, as already suggested in my first review, from my point of view the authors need to address my previous issues in their paper.

Response: During resubmission of the previous version of our manuscript [PONE-D-21-36646], we have answered to the first question in our ‘Response to Reviewers’, which may not have been adequate, and the suggested references have not been included by mistake. We apologize for the inadvertent omissions. Both the issues raised by the learned Reviewer have now been addressed in the manuscript with the inclusion of the suggested references.

Specifically, regarding the first question, the following lines have been inserted in the manuscript:

Line # 109: “All subjects enrolled in the study had blood glucose levels below 6.5 mmol/L since hyperglycemia also induces oxidative stress, and is common during ACS [17], to avoid false positive results.”

Line # 245: “Since hyperglycemia decreases regenerative potential of the myocardium [25], we excluded patients and controls with hyperglycemic random blood sugar levels from our investigation, to evaluate the association of albumin, fibrinogen and modified proteins with ACS independently from hyperglycemia.”

As for the second question, we are apologetic for failing to clarify the second issue both in our manuscript and ‘Response to Reviewers’. To address this important issue, we have included an extra column in Table 2 in the accompanying revised version [PONE-D-21-36646R1], to present the statistical findings of the univariate logistic regression analyses along with those of the multivariate regression data. Also, the following lines have been inserted in the manuscript:

Line # 297: “It is well established that hypertension damages the arteries by making them less elastic which decreases the flow of blood and oxygen to the heart; on the other hand, age can cause the development of additional risk factors such as obesity which may also affect the heart. Although there was a significant difference in age and hypertension between cases and controls, multivariate logistic regression analysis did not find age and hypertension to be significant risk factors for ACS after adjusting for other variables in this study. This could be because in our study population, age and hypertension were weaker risk factors compared to the other biochemical variables.”

To our understanding, since multivariate regression analysis considers more than one factor of independent variables that influence the variability of dependent variables, the conclusion drawn is more accurate, more realistic, and nearer to the real-life situation for our study population.

Reviewer #2: The authors adequately reply to all previous comments, and I am happy with the revised version, the manuscript is significantly improved

Reviewer #3: All comments have been addressed

7. PLOS authors have the option to publish the peer review history of their article (what does this mean?). If published, this will include your full peer review and any attached files.

If you choose “no”, your identity will remain anonymous but your review may still be made public.

Do you want your identity to be public for this peer review? For information about this choice, including consent withdrawal, please see our Privacy Policy.

Reviewer #1: No

Reviewer #2: Yes: Mohammed Ahmed Akkaif

Reviewer #3: No

[NOTE: If reviewer comments were submitted as an attachment file, they will be attached to this email and accessible via the submission site. Please log into your account, locate the manuscript record, and check for the action link "View Attachments". If this link does not appear, there are no attachment files.]

While revising your submission, please upload your figure files to the Preflight Analysis and Conversion Engine (PACE) digital diagnostic tool, https://pacev2.apexcovantage.com/. PACE helps ensure that figures meet PLOS requirements. To use PACE, you must first register as a user. Registration is free. Then, login and navigate to the UPLOAD tab, where you will find detailed instructions on how to use the tool. If you encounter any issues or have any questions when using PACE, please email PLOS at figures@plos.org. Please note that Supporting Information files do not need this step.
